# A targeted lipidomics approach to the study of eicosanoid release in synovial joints

**DOI:** 10.1186/ar3427

**Published:** 2011-07-27

**Authors:** Janny C de Grauw, Chris HA van de Lest, Paul René van Weeren

**Affiliations:** 1 Department of Equine Sciences, Faculty of Veterinary Medicine, Utrecht University, Yalelaan 114, 3584 CM, Utrecht, The Netherlands; 2 Department of Biochemistry and Cell Biology, Faculty of Veterinary Medicine, Utrecht University, Yalelaan 2, 3584 CM, Utrecht, The Netherlands

## Abstract

**Introduction:**

Articular tissues are capable of producing a range of eicosanoid mediators, each of which has individual biological effects and may be affected by anti-inflammatory treatment. We set out to develop and evaluate a high performance liquid chromatography-tandem mass spectrometry (HPLC-MS/MS) approach for the simultaneous analysis of multiple eicosanoid lipid mediators in equine synovial fluid (SF), and to illustrate its use for investigation of the *in vivo *effects of non-steroidal anti-inflammatory drug (NSAID) treatment.

**Methods:**

Synovial fluid samples were obtained from normal joints of 6 adult horses at baseline (0 hr) and at 8, 24 and 168 hours after experimental induction of transient acute synovitis, with horses treated once daily with oral NSAID (meloxicam, 0.6 mg/kg) or placebo. Following solid-phase extraction, SF lipid mediator quantitation was based on liquid chromatography-electrospray ionization-tandem mass spectrometry (LC-ESI-MS/MS) analysis, and results were compared between disease states using linear discriminant analysis (LDA) and analysis of variance (ANOVA) with multiple comparisons corrections.

**Results:**

Of a total of 23 mediators targeted, 14 could be reliably identified and quantified in SF samples based on detection of characteristic fragment ions at retention times similar to those of commercial standards. LDA analysis of baseline, 8, 24 and 168 hour synovial fluid samples revealed a separation of these groups into discrete clusters, reflecting dynamic changes in eicosanoid release over the course of synovitis. Prostaglandin (PG) E_2 _was significantly lower in NSAID vs. placebo treated samples at all time points; PGE_1_, 11-hydroxyeicosatetraenoic acid (11-HETE) and 13,14-dihydro-15keto PGF_2_α were reduced at 8 and 24 hours by NSAID treatment; while 15-HETE, 6-keto PGF_1_α, PGF_2_α, 13,14-dihydro-15keto PGE_2 _and thromboxane B_2 _(TXB_2_) were reduced at the 8 hour time point only. An interesting pattern was seen for Leukotriene B_4 _(LTB_4_), NSAID treatment causing an initial increase at 8 hours, but a significant reduction by 168 hours.

**Conclusions:**

The described method allows a comprehensive analysis of synovial fluid eicosanoid profiles. Eicosanoid release in inflamed joints as well as differences between NSAID treated and placebo treated individuals are not limited to PGE_2 _or to the early inflammatory phase.

## Introduction

Lipid mediators of inflammation play an important role in the local inflammatory response associated with inflammatory arthritides as well as orthopedic arthropathies [1]. Of these mediators, the E-series prostaglandins (most notably PGE_2_) are most noted in arthritis research for their pro-inflammatory and pro-nociceptive actions in synovial joints [2,3].

However, COX and LOX enzyme activity within the arachidonic acid cascade generates a range of eicosanoid mediators that have widely varying biological actions, including anti-inflammatory and pro-resolving effects [4,5]. In recent years, more light has been shed on the specific actions of individual eicosanoids in arthritis, and several of these (including PGE_2_) have emerged as janus-faced mediators with pro-inflammatory or anti-inflammatory effects depending effects, depending on concentration and receptor subtype engagement [6,7]. Reduction of PGE_2 _production is the classical mode of action of anti-inflammatory agents like non-steroidal anti-inflammatory drugs (NSAIDs) that are commonly used in medical management of (osteo)arthritis, and numerous studies have demonstrated a lower PGE_2 _concentration in synovial fluid (SF) following NSAID treatment [8-10]. However, by inhibition of COX activity, these drugs are likely not only to affect PGE_2 _production but also to interfere with the production of mediators with differential effects that are generated by the same enzymatic pathways. Indeed, in an early study, NSAID (naproxen) treatment tended to reduce not only PGE_2 _but also TXB_2 _and 6-keto PGF_1_α concentration in the SF of human patients with rheumatoid arthritis [8].

The investigation of the potential involvement of individual eicosanoids in disease states relies on sensitive and specific assays to measure these products in biological fluids. While antibody-based assays for individual eicosanoids are commonly employed, these suffer from cross-reactivity issues and may produce misleading results in complex biological samples [11]. Moreover, they can be used for analysis of only one metabolite at a time, restricting the amount of biological information obtained as SF sample volume tends to be a limiting factor.

In this report, we describe the application of recently developed high-performance liquid chromatography-tandem mass spectrometry (HPLC-MS/MS) mediator lipidomics techniques to the study of eicosanoid release in equine synovial joints. To evaluate the relative abundance of these lipid mediator species in normal and inflamed joints and investigate the effects of COX inhibition on eicosanoid profiles, we performed a longitudinal study of SF lipid mediator composition in healthy horses over the course of experimentally induced transient synovitis with and without oral NSAID treatment.

## Materials and methods

5(S)-hydroxyeicosatetraenoic acid (HETE), 8(S)-HETE, 11(S)-HETE, 12(S)-HETE, and 15(S)-HETE; leukotriene D_4 _(LTD_4_), LTE_4_; LTB_4_; 5(S)6(R)15(S)-lipoxin A_4 _(LXA_4_); prostaglandin E_1 _(PGE_1_), 6-keto prostaglandin F_1_α (6-keto PGF_1_α), prostaglandin D_2 _(PGD_2_), prostaglandin E_2 _(PGE_2_), prostaglandin F_2_α (PGF_2_α), 11β-prostaglandin F_2_α (11β-PGF_2_α), prostaglandin F_2_β (PGF_2_β), prostaglandin J_2 _(PGJ_2_), 15-deoxy-Δ12,14-prostaglandin J_2 _(15-deoxy-Δ12,14-PGJ_2_), thromboxane B_2 _(TXB_2_), 13,14-dihydro-15-keto PGF_2_α, 13,14-dihydro-15-keto PGE_2_, 13,14-dihydro-15-keto PGD_2_, and 16,16-dimethyl PGF_2_α were purchased from Cayman Chemical Company (Ann Arbor, MI, USA). HPLC-grade solvents (acetonitrile and methanol) were from Biosolve (Valkenswaard, The Netherlands), and glacial acetic acid and all other chemicals used for sample extraction and preparation were from Sigma-Aldrich (St. Louis, MO, USA). Solid-phase extraction columns (LiChrolut RP-18; 100-mg column bed) were purchased from Merck (Darmstadt, Germany).

### Preparation of standards and calibration lines

Stock standard solutions were prepared in ethanol (100 ng/μL) and stored in amber vials at -80°C under N_2_. Calibration lines were prepared by diluting the appropriate stock solutions to final concentrations of 100, 50, 25, 10, 5, 2, and 1 pg/μL. The internal standard (IS) (16,16-dimethyl-PGF_2_α) was prepared in ethanol (2 ng/μL) and was added to all composite standards at a final concentration of 100 pg/μL. Chromatograms for standards were used to establish characteristic retention times (RTs) of each compound, while the calibration lines were used to verify that the MS signal was linear for all analytes over this range. The peak-area ratios of each analyte to IS (16,16-dimethyl-PGF_2_α) were calculated and plotted against the concentration of the calibration standards. Calibration lines were calculated by the least squares linear regression method.

### Sample collection and storage

SF samples were obtained from a previously reported cross-over study of NSAID versus placebo treatment in an equine lipopolysaccharide (LPS)-induced transient synovitis model [9]. All experimental procedures were preapproved by the Utrecht University institutional Ethics Committee on Animal Experimentation. In short, six healthy adult warmblood horses were subjected to two episodes of experimental synovitis, once in the right and once in the left middle carpal joint, with a 2-week washout period in between. During each experimental period, horses were randomly assigned to receive oral NSAID treatment (meloxicam, 0.6 mg/kg; Boehringer Ingelheim Vetmedica GmbH, Ingelheim am Rhein, Germany) or placebo treatment (the same oral suspension minus the active substance) starting at t = 2 hours after LPS and at 24-hour intervals thereafter (26, 50, 74, 98, 122, and 146 hours after LPS) for a total of seven treatments. In each experimental period, SF samples were aspirated at baseline (t = 0, just prior to LPS injection) and 8, 24, and 168 hours after LPS. Samples were centrifuged at 10,000*g *immediately after collection, and supernatants were aliquotted and transferred to -80°C within 30 minutes; samples were stored at -80°C awaiting extraction.

### Sample preparation

SF aliquots (300 μL) were thawed on ice. IS (20 μL of a 200 pg/μL solution of 16,16-dimethyl PGF_2_α) was added to each sample. Samples were diluted with 1.5 mL of 15% (vol/vol) methanol in 0.1% (vol/vol) formic acid and 0.002% (vol/vol) butylated hydroxytoluene (BHT) (an anti-oxidant), incubated on ice for 10 minutes, and then centrifuged at 10,000*g *for 15 minutes at 4°C to remove any precipitated proteins. The resulting clear supernatants were decanted and kept on ice. Pellets were washed with a further 1.2 mL of 15% (vol/vol) methanol in 0.1% (vol/vol) formic acid and 0.002% (vol/vol) BHT and again were centrifuged for 10 minutes at 10,000*g *and 4°C, after which this supernatant was added to that previously collected, making a total sample volume of 3 mL.

This sample was applied to RP-18 solid-phase extraction columns (100 mg) that had been preconditioned with 1 mL of acetone followed by 1 mL of 15% (vol/vol) methanol in 0.1% (vol/vol) formic acid. The columns were then washed with 1 mL of 15% (vol/vol) methanol in 0.1% (vol/vol) formic acid, 2 × 1 mL of water, and 1 mL of hexane. Lipid mediators were eluted into amber vials containing silanized glass inserts using 3 × 250 μL volumes of ethylacetate. The eluate was evaporated under nitrogen; the residue was dissolved in 40 μL of ethanol, flushed with nitrogen, and stored at -80°C awaiting HPLC-MS/MS analysis.

### High-performance liquid chromatography-tandem mass spectrometry analysis

HPLC-MS/MS analysis was performed on a PerkinElmer LC200 HPLC system (PerkinElmer, Waltham, MA, USA) coupled to an electrospray ionization (ESI) linear ion trap quadrupole (4000 QTRAP) mass spectrometer (Applied Biosystems, Nieuwerkerk aan den IJssel, The Netherlands). The instrument was operated in the negative ionization mode. For all experiments, the ion source voltage was -4,500 V and the source temperature was 350°C. Multiple reaction monitoring (MRM) of 26 mass-to-charge (m/z) transitions was used for compound quantification, and declustering potential and collision energy (using nitrogen as collision gas) were empirically optimized for each compound (Table 1).

**Table 1:** Multiple reaction monitoring transitions for liquid chromatography-tandem mass spectrometry assay of eicosanoids

Compound	MRM, m/z	Collision energy, eV	Declustering potential
PGE_1_	353 → 317	-30	-80
6-keto PGF_1_α	369 → 163	-35	-100
PGD_2_	351 → 271	-25	-40 and -80
PGE_2_	351 → 271	-25	-40 and -80
	351 → 175	-30	-80
PGF_2_α	353 → 193	-35	-90
11β-PGF_2_α	353 → 309	-20	-60
PGF_2_β	353 → 309	-25	-40
PGJ_2_	333 → 189	-25	-40
	333 → 271	-25	-80
15-deoxy-Δ-12,14 PGJ_2_	315 → 271	-20	-90
13,14-dihydro-15-keto PGD_2_	351 → 207	-27	-80
13,14-dihydro-15-keto PGE_2_	351 → 333	-17	-80
13,14-dihydro-15-keto PGF_2_α	353 → 113	-40	-100
TXB_2_	369 → 195	-23	-80
	369 → 169	-25	-90
LTB_4_	335 → 195	-20	-100
5(S)-HETE	319 → 115	-20	-40
8(S)-HETE	319 → 155	-20	-80
11(S)-HETE	319 → 167	-20	-80
12(S)-HETE	319 → 179	-20	-80
15(S)-HETE	319 → 175	-20	-80
LTD_4_	495 → 177	-30	-100
LTE_4_	438 → 333	-25	-100
LXA_4_	351 → 235	-20	-80
16,16-dimethyl PGF_2_α (IS)	381 → 319	-35	-100

eV, electron volts; HETE, hydroxyeicosatetraenoic acid; IS, internal standard; LT, leukotriene; LX, lipoxin; MRM, multiple reaction monitoring; m/z, mass/charge; PG, prostaglandin; TX, thromboxane.

Chromatographic analysis was performed on a C18 column (Luna, 2.5 μm 100 × 3 mm; Phenomenex, Torrance, CA, USA). The injection volume was 10 μL, and the flow rate 0.2 mL/minute. The column was maintained at ambient temperature. The analysis was performed by using a linear gradient obtained by mixing solvents A (0.02% (vol/vol) glacial acetic acid in water) and B (0.02% (vol/vol) glacial acetic acid in acetonitrile) as follows: from 0 to 1 minute: 80% A, from 1 to 17 minutes: 63% A; from 17 to 18 minutes: 52% A; from 18 to 23 minutes: 100% B; and from 24 to 25 minutes: 80% A.

### Data analysis

Automatic peak detection and integration were performed by using the XCMS software package [12]. Data were processed by using XCMS version 1.22.1 running under R version 2.11.0. The signal-to-noise ratio for peak detection was set to 10. Zero values, in samples with missing peaks, were prevented by forced integration at the calculated expected RT of the peak. Linear discriminant analysis (LDA) was performed by using MarkerView software (MarkerView 1.1.0.7; Applied Biosystems, Foster City, CA, USA) and visualized graphically using GraphPad (GraphPad Prism version 5.2 for Windows; GraphPad Software, San Diego, CA, USA). Significance was tested by using analysis of variance and *t *tests with Welch's correction for unequal variances. Since relatively large numbers of peaks were tested simultaneously, small *P *values occurred by chance and false-positives were expected; these were corrected for by using the R package MULTTEST [13,14].

## Results

### Extraction efficiency

Analyte recovery was estimated by comparing the peak area of the IS added to each SF sample prior to extraction, and the peak-area value was obtained in the pure (that is, unextracted) IS solution. In both conditions, a total of 1,000 pg of 16,16-dimethyl PGF_2_α was brought on-column assuming 100% extraction efficiency. Mean (± standard deviation) recovery of IS in SF samples (*n *= 48) was 69.5% ± 10.8% (range of 48.7% to 90.4%).

### Linearity

Calibration curves of standards showed excellent linearity over a concentration range of 1 to 100 pg/μL (corresponding to 10 to 1,000 pg on-column), and correlation coefficients were greater than 0.99 for all analytes except for 8-HETE (*r *= 0.988) and 5-HETE (*r *= 0.969). See Figure S1 of Additional file 1.

### Eicosanoid identification and quantitation

**Figure 1:**
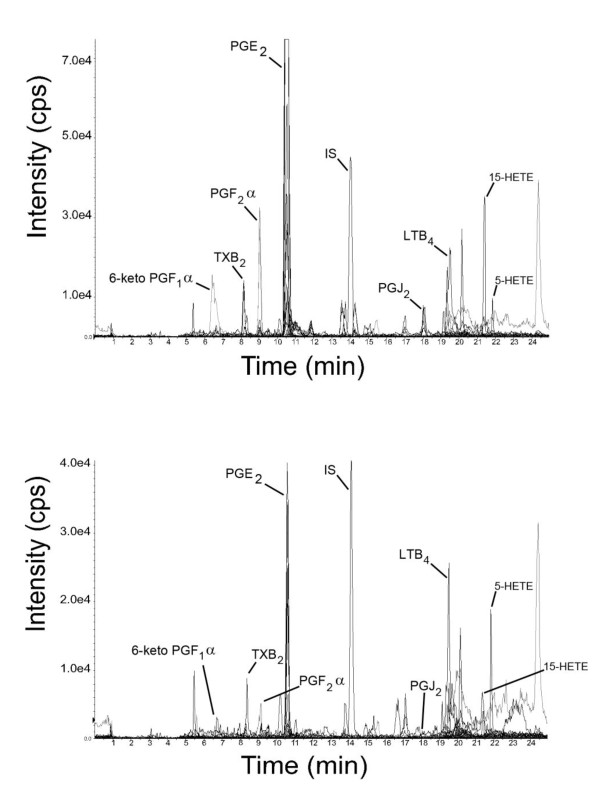
**Representative reconstructed chromatograms of synovial fluid extracts**. Total ion count (counts per second, or cps) versus time in a placebo-treated sample (top panel) at t = 8 hours after lipopolysaccharide and an 8-hour non-steroidal anti-inflammatory drug-treated sample (bottom panel) from the same subject. Note the difference in scales on the y-axes between top and bottom panels. HETE, hydroxyeicosatetraenoic acid; IS, internal standard; LT, leukotriene; PG, prostaglandin; TX, thromboxane.

**Figure 2:**
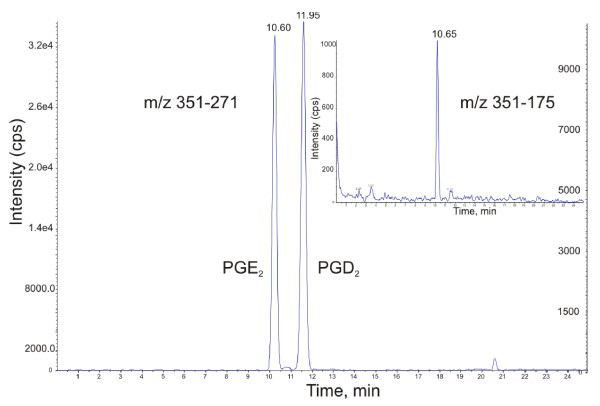
**Extracted ion chromatogram of mass transition 351→271 in an 8-hour synovitis sample**. The separate peaks show excellent chromatographic resolution of geometrical isomers prostaglandin E_2 _(PGE_2_) and PGD_2_. The low-abundant peak at 351→175 (inset) coincides with the first peak of the main trace, confirming the identity of this analyte as PGE_2 _rather than PGD_2_. Note the difference in scales of the left y-axis (showing ion count for PGE_2_) and the right y-axis (PGD_2_). cps, counts per second; m/z, mass/charge.

Reconstructed chromatograms of SF samples showed adequate peak resolution (Figure 1), and inclusion of more than one MRM transition for an analyte proved to be a useful adjunct to chromatographic resolution for analyte identification (Figure 2). Peaks were integrated and RTs were compared with those of commercially available standards of the compound of interest. Only peaks with the correct combination of m/z transition and RT were considered to be positively identified as the analyte of interest and were subsequently quantitated with reference to the standard curve of the particular analyte (Figure S1 of Additional file 1).

**Figure 3:**
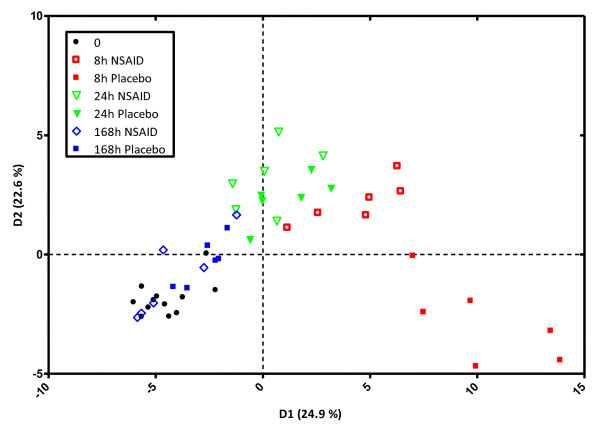
**Linear discriminant analysis showing discriminant 2 versus 1 of synovial fluid eicosanoid profiles**. Samples were taken at four separate time points (0, 8, 24, and 168 hours) from individuals treated with non-steroidal anti-inflammatory drug (NSAID) (*n *= 6) or placebo (*n *= 6) over the course of transient acute synovitis. Linear discriminant analysis finds a linear combination of features ('discriminants') that characterize or separate two or more classes of subjects (in this case, samples). Seven classes were predefined: 0 hours (baseline, no treatment administered yet), 8 hours of placebo, 8 hours of NSAID, 24 hours of placebo, 24 hours of NSAID, 168 hours of placebo, and 168 hours of NSAID. The x- and y-axes denote discriminant 1 (D1) and discriminant 2 (D2), respectively. D1 has a slightly higher weighing factor than discriminant 2 (D2) as D1 explains 24.9% of the observed variance between classes and D2 22.6%. The distance between groups in this plot denotes the degree of dissimilarity between samples belonging to each group.

**Figure 4:**
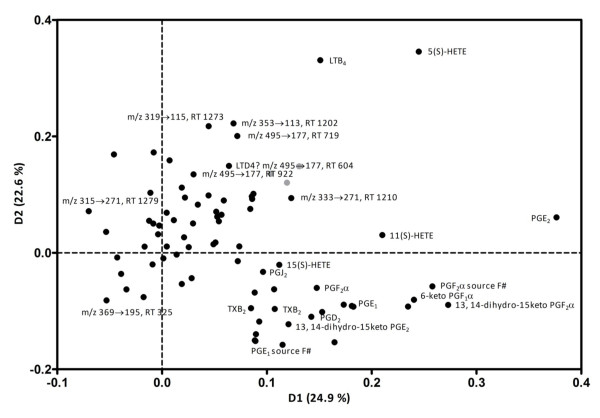
**Loading plot pertaining to linear discriminant analysis of synovial fluid eicosanoid profiles**. Samples were taken at four separate time points (0, 8, 24, and 168 hours) from individuals treated with non-steroidal anti-inflammatory drug (*n *= 6) or placebo (*n *= 6) over the course of transient acute synovitis. The loading plot shows all detected mass transitions, highlighting those that contributed most to the observed differences (spatial separation) between samples over time and with treatment. Points denoting mass transitions that are furthest away from the intercept of both axes contributed most to the differences between samples (labeled with mediator name if positively identified or with mass transition and retention time when the identity could not be confirmed by reference to commercial standards), whereas those close to the intercept depict mass transitions that were common to most samples. HETE, hydroxyeicosatetraenoic acid; LT, leukotriene; m/z, mass/charge; PG, prostaglandin; TX, thromboxane.

**Figure 5:**
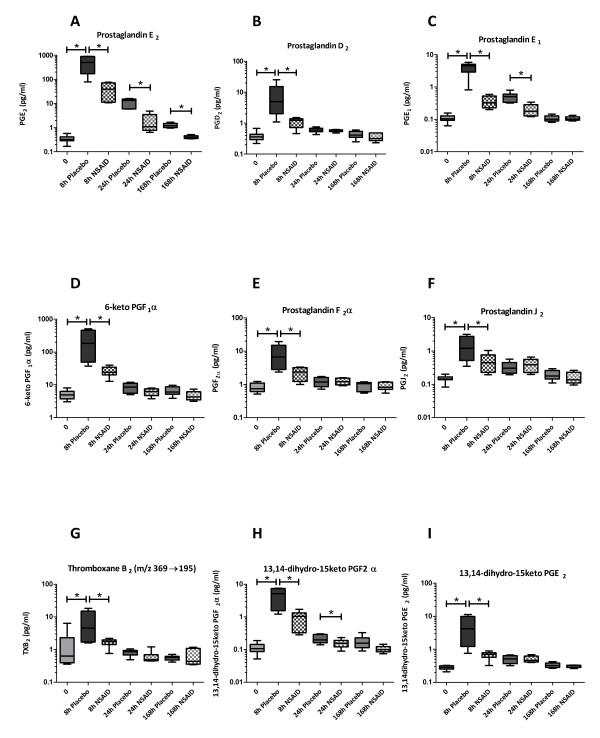
**Concentration of prostanoid species in synovial fluid over the course of lipopolysaccharide-induced synovitis**. Horses were treated with a non-steroidal anti-inflammatory drug (NSAID) (meloxicam, 0.6 mg/kg once a day by mouth; *n *= 6) or placebo (*n *= 6) starting at 2 hours after lipopolysaccharide injection for a total of seven treatments. Boxes depict median and interquartile range; whiskers denote minimum and maximum values. *P < 0.05. PG, prostaglandin; TX, thromboxane.

Unidentified peaks (that is, m/z transitions detected at non-characteristic RTs for the analyte of interest) were evaluated for alternative processes that might generate such peaks, such as (source) fragmentation of closely related mediators eluting at that RT. All monitored m/z transitions were included in LDA of NSAID- versus placebo-treated samples at each time point (Figure 3); this statistical method analyzes complex data by graphically presenting the degree of (dis)similarity between samples belonging to the same or to different groups on the basis of variables measured in each sample (in this case, m/z transitions). The corresponding loading plot (Figure 4) shows which fragments contributed most to the observed difference (that is, spatial separation) between samples belonging to different groups; those mediators that are furthest from the intercept of both axes (the 0 distance point) contribute most to this distinction (that is, PGE_2_, LTB_4_, 5-HETE, 11-HETE, 6-keto PGF_1_α, PGF_2_α, 13,14-dihydro-15-keto PGF_2_α, and TXB_2_). Concentration profiles of individual eicosanoids in SF over the course of synovitis with or without NSAID treatment are shown in Figures 5 (prostanoids) and 6 (HETEs and LTB_4_).

**Figure 6:**
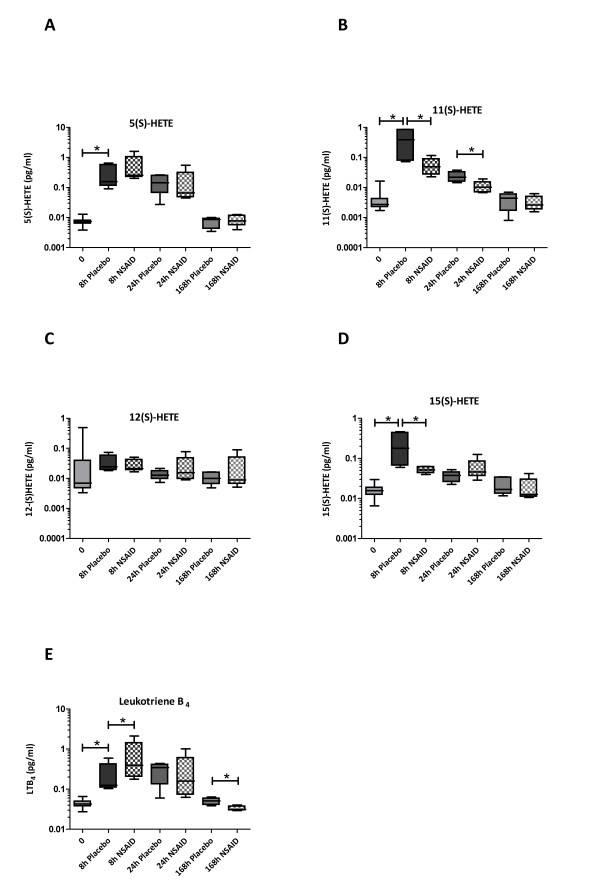
**Synovial fluid hydroxyeicosatetraenoic acid (HETE) (a-d) and leukotriene B_4 _(LTB_4_) (e) concentrations during lipopolysaccharide (LPS)-induced synovitis**. Horses were treated with a non-steroidal anti-inflammatory drug (NSAID) (meloxicam, 0.6 mg/kg once a day by mouth; *n *= 6) or placebo (*n *= 6) starting at 2 hours after LPS injection for a total of seven treatments. Individual HETEs respond differentially to LPS and NSAID treatment. Boxes depict median and interquartile range; whiskers denote minimum and maximum values. *P < 0.05.

## Discussion

In this report, we describe the application of mediator lipidomics techniques to the study of SF eicosanoid profiles in normal and inflamed equine joints. Furthermore, we illustrate the use of the developed LC-ESI-MS/MS analysis to investigate the effects of NSAID treatment in acute synovitis.

We identified and quantitated 14 individual eicosanoids in SF extracts by using MRM. The MRM transitions used to identify individual compounds in this study were confirmed by literature sources [15-23]; unfortunately, as previously outlined by Murphy and colleagues [20], many eicosanoids have very similar or even identical (isomeric) chemical structures and therefore a single m/z transition may not be as specific to individual compounds as desired. Future experiments employing information-dependent acquisition in combination with enhanced product ion detection settings could be used to enhance analyte identification [24]. Alternatively, the use of more than one m/z transition per compound (as was done in the present case for TXB_2 _and PGE_2_) could aid in definitive identification.

The recovery of analytes in this study was estimated by comparing peak areas of the IS in spiked and extracted SF samples with the corresponding standard solution analyzed without extraction. Although this provides an indication of analyte loss over the extraction procedure, it does not account for possible differences in recovery or degradation between individual analytes. While it is certainly not uncommon to use only one IS in studies of multiple analytes [15,21,25], it would be preferable to use stable isotope-labeled standards for each analyte under investigation or to use one such labeled standard per class of mediators targeted [24]. However, the 16,16-dimethyl PGF_2_α we employed does combine several advantages for use as an IS in the current application: It elutes at an RT close to that of many analytes of interest, it shows good stability at room temperature and at -20°C and -80°C (de Grauw, unpublished observations), and it is not normally found in SF and is not known to have a biological function in synovial joints.

In addition to rapid enzymatic conversion and degradation of lipid mediators *in vivo*, the reliable analysis of eicosanoids in body fluids may be hampered by *ex vivo *degradation of labile species [24]. This was recently demonstrated for PGD_2_, which was shown to be far more susceptible to chemical decomposition at room temperature and at -20°C than PGE_2 _[23]. Hence, relative quantities of these mediators in extracted samples may also reflect selective degradation of one over the other, and absolute levels should be interpreted with caution; the same might be true for other eicosanoids, the stability of which has not been exhaustively addressed. For instance, the current extraction procedure and HPLC solvent system are not optimized for quantitative detection of cysteinyl LTs [25].

We positively identified 14 eicosanoids in SF extracts and found that the concentrations of many of these were significantly elevated in inflamed joints compared with normal (baseline) values (Figures 5 and 6). As LPS induces marked influx of leukocytes (predominantly neutrophils [9]) into the joint space, eicosanoid species detected may partly reflect release by infiltrating cells rather than release from articular sources; however, articular cartilage and, especially, synovial fibroblasts are apt producers of a great number of these mediators [17,26,27], and therefore having the means to detect these will aid in future studies of spontaneous disease.

SF eicosanoid profiles changed dramatically upon synovitis induction, as illustrated by the LDA plots showing marked separation between samples taken at different time points. Release of individual prostanoids, HETEs, and LTB_4 _over the course of transient synovitis did not reveal marked temporal differences between these classes of mediators, although there was a trend toward early response of prostanoids versus a somewhat more protracted response of HETEs and LTB_4_. PGE_1 _and PGE_2 _as well as PGJ_2 _(which is an intermediate breakdown product of PGD_2 _and which is known to have anti-inflammatory properties [28,29]) showed a more prolonged elevation over the first 24 hours of synovitis than other prostanoids. Although our method quantitatively detected 15-deoxy-Δ12,14-PGJ_2 _in stock standards and in spiked SF, this anti-inflammatory PG was not detected in SF extracts and hence we cannot comment on its temporal release pattern. The same was true for LXA_4_, another endogenous anti-inflammatory and pro-resolving mediator [5]. Some of these species may have escaped detection because of rapid enzymatic conversion or chemical instability as noted above; thus, the precise release kinetics of pro- versus anti-inflammatory eicosanoid species in acute synovitis will need to be addressed in future studies that include even earlier sampling time points or alternative extraction steps or both.

The observed differences between NSAID- and placebo-treated samples clearly demonstrate that the effects of COX inhibitors on synovial eicosanoid release are not limited to PGE_2 _reduction. Concentrations of many other prostanoids, including PGE_1_, PGD_2_, PGJ_2_, PGF_2_α, 6-keto PGF_1_α, and TXB_2_, were also significantly lower in NSAID- versus placebo-treated SF samples, particularly in the acute phase of synovitis, and this agrees with and extends previous findings in SF of human subjects treated with naproxen [8]. The observed reduction in PGE_2 _and 6-keto PGF_1_α (the stable main metabolite of prostacyclin) with NSAID treatment is likely to contribute to analgesic efficacy [30]. Inhibition of PGE_2 _and PGE_1 _production could limit the negative feedback of these two mediators on matrix metalloproteinase (MMP) production by synovial fibroblasts [28]; however, as meloxicam itself inhibits synovial MMP activity [9], this will not have clinical implications. The consequences of TXB_2_, PGD_2_, and PGJ_2 _inhibition are harder to predict because their roles in arthritis have been less well studied.

Our results for LTB_4 _and 5-, 12-, and 15-HETE are interesting because these mediators are all products of the LOX pathway but proved to be differentially affected by NSAID treatment. Both LTB_4 _and 5-HETE are downstream products of 5-LOX, 12-HETE is produced through 12-LOX action, and 15-HETE is produced by 15-LOX (while both 11- and 15-HETE can also be produced by COX [31]). As seen in Figure 6, 12-HETE concentration did not change at all over the course of transient synovitis, whereas the concentration of 15-HETE was significantly elevated in the acute phase and reduced by NSAID treatment. Interestingly, the concentration of LTB_4 _was significantly higher at 8 hours in SF of NSAID-treated versus placebo-treated horses, and 5-HETE showed a similar trend. A transient increase in LTB_4 _release has also been found in cultured synovial membrane and cartilage explants treated with certain COX inhibitors [28,32] and has been suggested to be due to 'shunting' of arachidonic acid away from COX-mediated PG production toward LOX-mediated LT production [28]. However, our findings suggest that this is an oversimplification since such a general shunt would have resulted in elevated concentrations of all LOX-generated mediators rather than some of them. Perhaps more likely, different LOX isoforms or additional enzymes (or both) that are located within the arachidonic acid cascade and that act upon intermediate metabolites (for example, phospholipid hydroperoxide glutathione peroxidase) may be differentially affected by NSAID treatment. Interestingly, LTB_4 _elevation was no longer observed at later time points, and, at 168 hours, meloxicam actually reduced LTB_4 _concentration. The biological or clinical implications of this transient rise in LTB_4 _upon therapeutic COX inhibition remain to be established [28] and are likely to be complex; while LTB_4 _plays a crucial role in driving inflammatory arthritis [33], it may also act to rescue COX-generated mediator production important to resolution of inflammation [34].

Altogether, this study represents a first attempt at simultaneous quantification of more than 20 lipid mediators in SF samples. Despite the more complex data analysis and inherent difficulty in identification of multiple closely related analytes in complex biological samples, the ESI-LC-MS/MS method we employed shows obvious advantages over enzyme-linked immunosorbent assay-based techniques that require relatively large volumes of SF for detection of one selected mediator at a time, while unaccounted-for analyte loss during sample extraction and cross-reactivity between analytes may provide misleading results [15,24]. The current approach allows high-throughput screening of SF samples by using an IS for estimation of extraction efficiency and requires only 300 μL of SF to analyze a much wider spectrum of eicosanoids, thus enabling the detection of mediators and treatment effects that otherwise would have escaped attention.

## Conclusions

We report the application of a sensitive HPLC-MS/MS technique for the simultaneous detection of more than 20 eicosanoids in SF. Extraction efficiency was deemed adequate, and the method showed good linearity and sensitivity. The application of this method to SF samples from horses with experimentally induced synovitis treated with NSAID or placebo confirmed local release of many more eicosanoids than PGE_2 _alone over the course of transient synovitis and revealed differential effects of NSAID treatment on several of these mediators: PGE_2 _was significantly lower in NSAID- versus placebo-treated samples at all time points; PGE_1_, 11-HETE, and 13,14-dihydro-15-keto PGF_2_α were reduced throughout the acute phase (8 and 24 hours) by NSAID treatment; whereas 15-HETE, 6-keto PGF_1_α, PGF_2_α, 13,14-dihydro-15-keto PGE_2_, and TXB_2 _were reduced at the earliest time point only. An interesting pattern was seen for LTB_4_, in which NSAID treatment caused an initial increase at 8 hours but a significant reduction at 168 hours.

## Abbreviations

BHT: butylated hydroxytoluene; ESI: electrospray ionization; HETE: hydroxyeicosatetraenoic acid; HPLC: high-performance liquid chromatography; IS: internal standard; LC: liquid chromatography; LDA: linear discriminant analysis; LPS: lipopolysaccharide; LT: leukotriene; LX: lipoxin; MMP: matrix metalloproteinase; MRM: multiple reaction monitoring; MS: mass spectrometry; m/z: mass/charge; NSAID: non-steroidal anti-inflammatory drug; PG: prostaglandin; RT: retention time; SF: synovial fluid; TX: thromboxane.

## Competing interests

The authors declare that they have no competing interests.

## Authors' contributions

JCdG carried out the sample collection and extractions, participated in method optimization, and drafted the manuscript. CHAvdL carried out the HPLC-MS/MS optimization and data analysis and performed the statistical analysis. PRvW participated in the design and coordination of the study and helped to draft the manuscript. All authors read and approved the final manuscript.

## Supplementary Material

Additional file 1 **Calibration lines - Standard curve equations and correlation coefficients for LC-ESI-MS/MS analysis of eicosanoid standards**. Calibration lines for liquid chromatography-electrospray ionization-tandem mass spectrometry (LC-ESI-MS/MS) analysis were prepared by diluting stock solutions to final concentrations of 100 pg/μL, 50 pg/μL, 25 pg/μL, 10 pg/μL, 5 pg/μL, 2 pg/μL and 1 pg/μL. The internal standard (IS; 16,16-dimethyl prostaglandin F_2_α) was prepared in ethanol (2 ng/μL) and added to all composite standards at a final concentration of 100 pg/μL. Click here for file
