# Supplementary material for: A targeted lipidomics approach to the study of eicosanoid release in synovial joints
Source: Arthritis Res Ther. 2011 Jul 27;13(4):R123. doi: 10.1186/ar3427 (PMC3239362; doi:10.1186/ar3427)
Supplement: Additional file 1 — Calibration lines - Standard curve equations and correlation coefficients for LC-ESI-MS/MS analysis of eicosanoid standards. Calibration lines for liquid chromatography-electrospray ionization-tandem mass spectrometry (LC-ESI-MS/MS) analysis were prepared by diluting stock solutions to final concentrations of 100 pg/μL, 50 pg/μL, 25 pg/μL, 10 pg/μL, 5 pg/μL, 2 pg/μL and 1 pg/μL. The internal standard (IS; 16,16-dimethyl prostaglandin F2α) was prepared in ethanol (2 ng/μL) and added to all composite standards at a final concentration of 100 pg/μL. [file ar3427-S1.PDF]

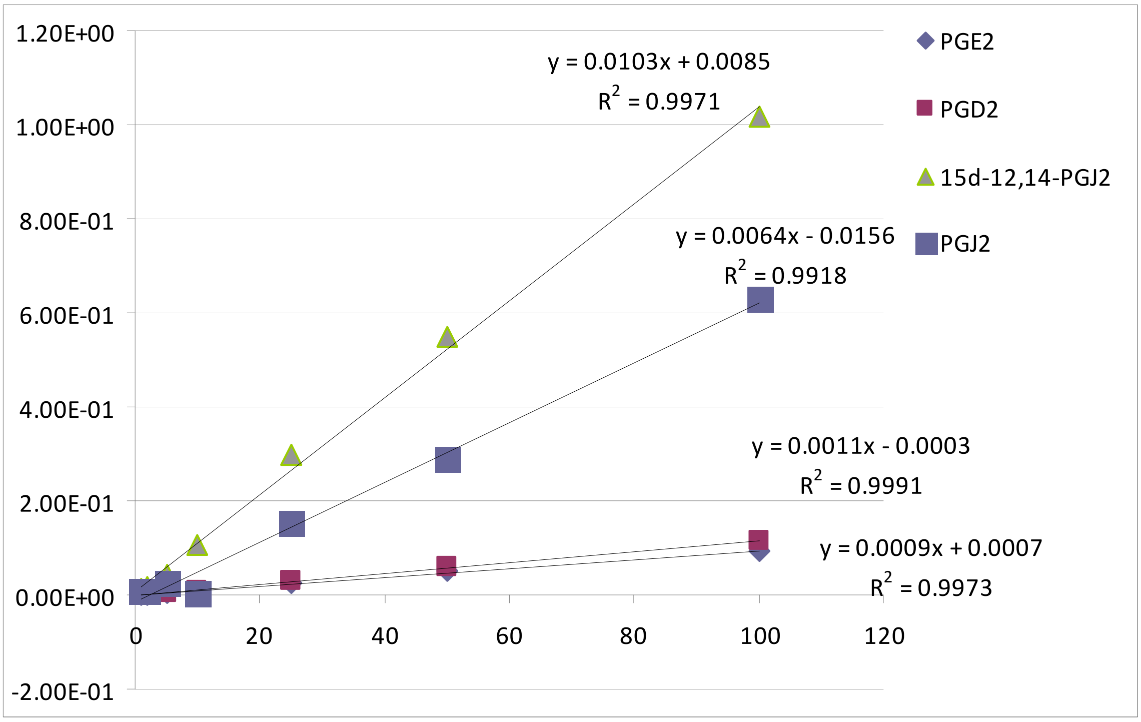
1.20E+00
1.00E+00
8.00E-01
6.00E-01
4.00E-01
2.00E-01
0.00E+00
-2.00E-01
0
20
40
60
80
100
120
y = 0.0103x + 0.0085
R^2 = 0.9971
y = 0.0064x - 0.0156
R^2 = 0.9918
y = 0.0011x - 0.0003
R^2 = 0.9991
y = 0.0009x + 0.0007
R^2 = 0.9973
PGE2
PGD2
15d-12,14-PGJ2
PGJ2

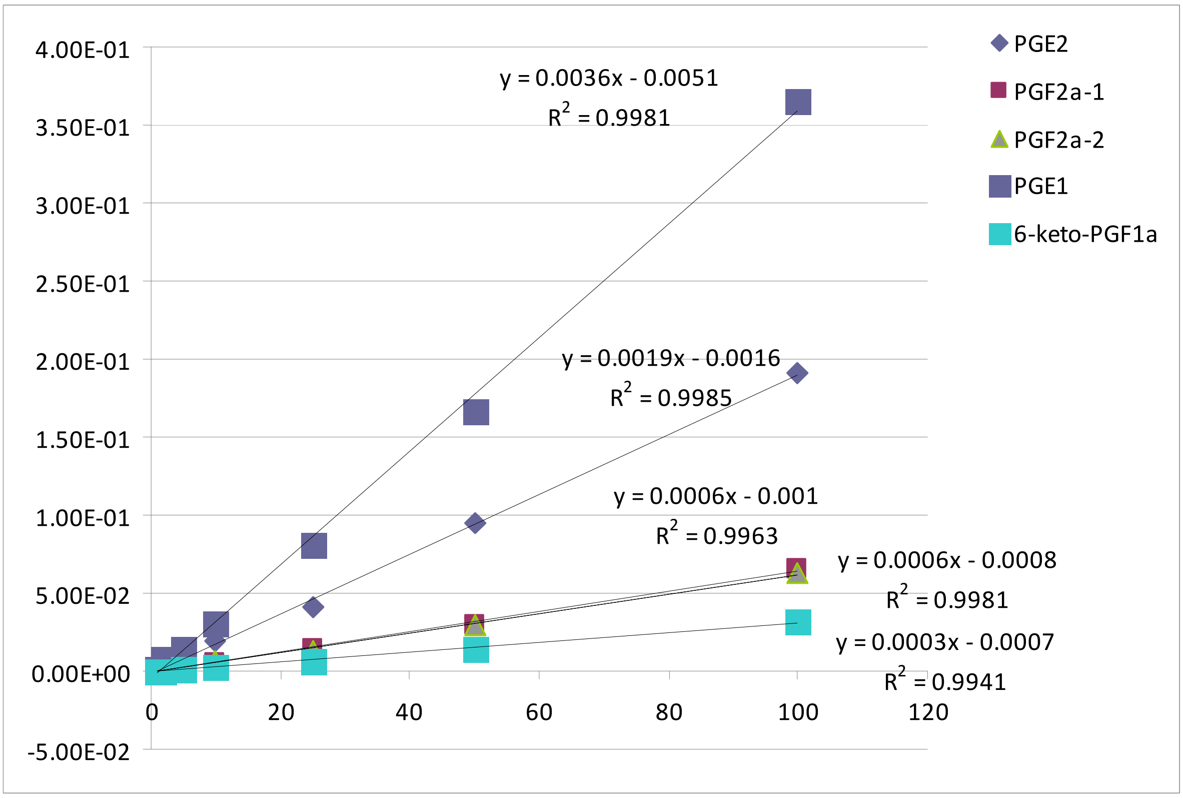
4.00E-01
3.50E-01
3.00E-01
2.50E-01
2.00E-01
1.50E-01
1.00E-01
5.00E-02
0.00E+00
-5.00E-02
0
20
40
60
80
100
120
y = 0.0036x - 0.0051
R^2 = 0.9981
y = 0.0019x - 0.0016
R^2 = 0.9985
y = 0.0006x - 0.001
R^2 = 0.9963
y = 0.0006x - 0.0008
R^2 = 0.9981
y = 0.0003x - 0.0007
R^2 = 0.9941
PGE2
PGF2a-1
PGF2a-2
PGE1
6-keto-PGF1a

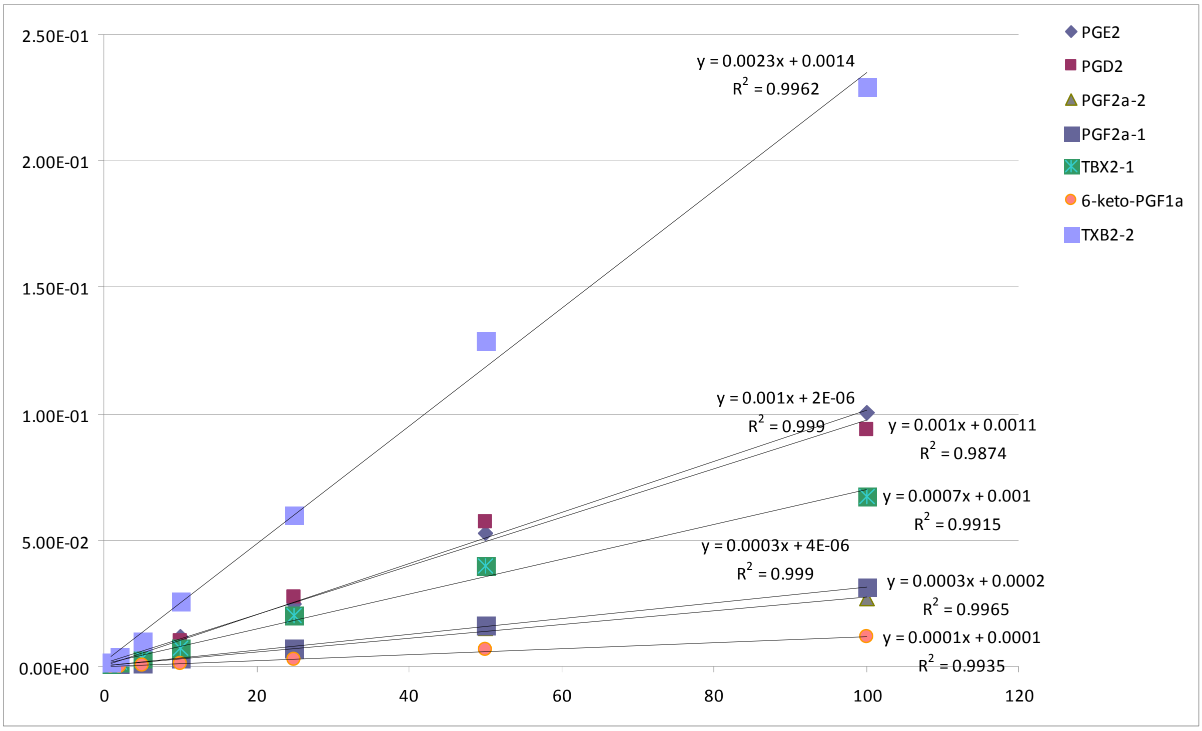

2.50E-01
2.00E-01
1.50E-01
1.00E-01
5.00E-02
0.00E+00
0
20
40
60
80
100
120
PGE2
PGD2
PGF2a-2
PGF2a-1
TBX2-1
6-keto-PGF1a
TXB2-2
y = 0.0023x + 0.0014
R2 = 0.9962
y = 0.001x + 2E-06
R2 = 0.999
y = 0.001x + 0.0011
R2 = 0.9874
y = 0.0007x + 0.001
R2 = 0.9915
y = 0.0003x + 4E-06
R2 = 0.999
y = 0.0003x + 0.0002
R2 = 0.9965
y = 0.0001x + 0.0001
R2 = 0.9935

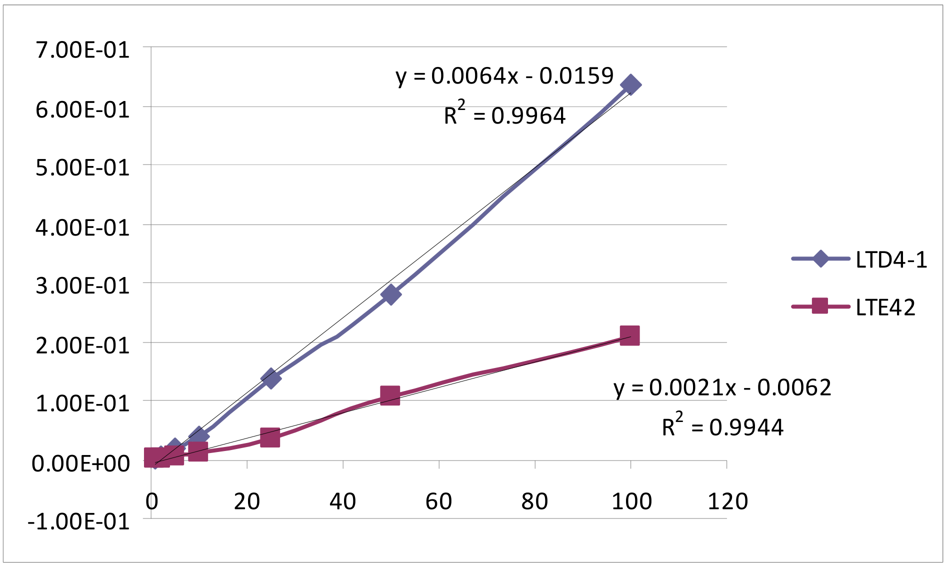

7.00E-01
6.00E-01
5.00E-01
4.00E-01
3.00E-01
2.00E-01
1.00E-01
0.00E+00
-1.00E-01
0
20
40
60
80
100
120
y = 0.0064x - 0.0159
R2 = 0.9964
y = 0.0021x - 0.0062
R2 = 0.9944
LTD4-1
LTE42

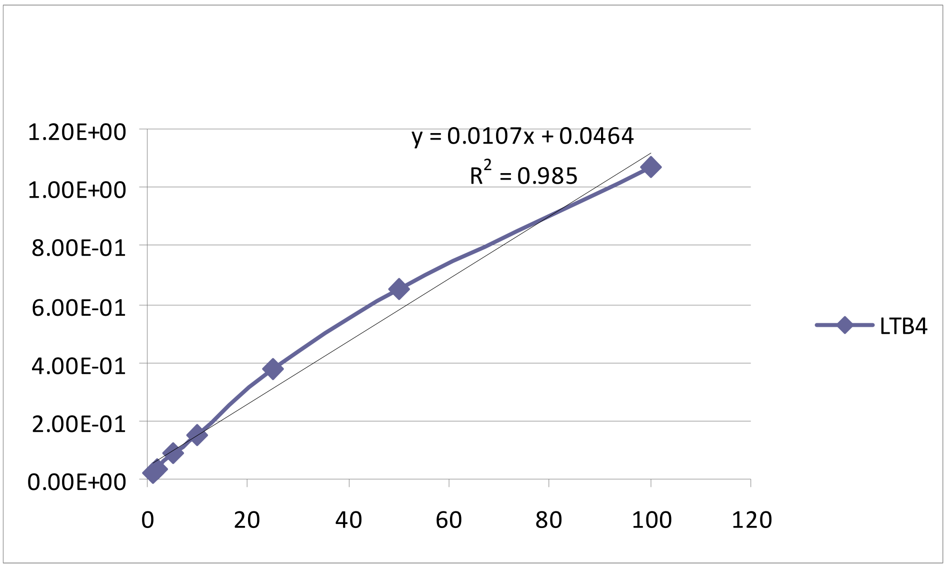

y = 0.0107x + 0.0464
R^2 = 0.985
1.20E+00
1.00E+00
8.00E-01
6.00E-01
4.00E-01
2.00E-01
0.00E+00
0
20
40
60
80
100
120
LTB4

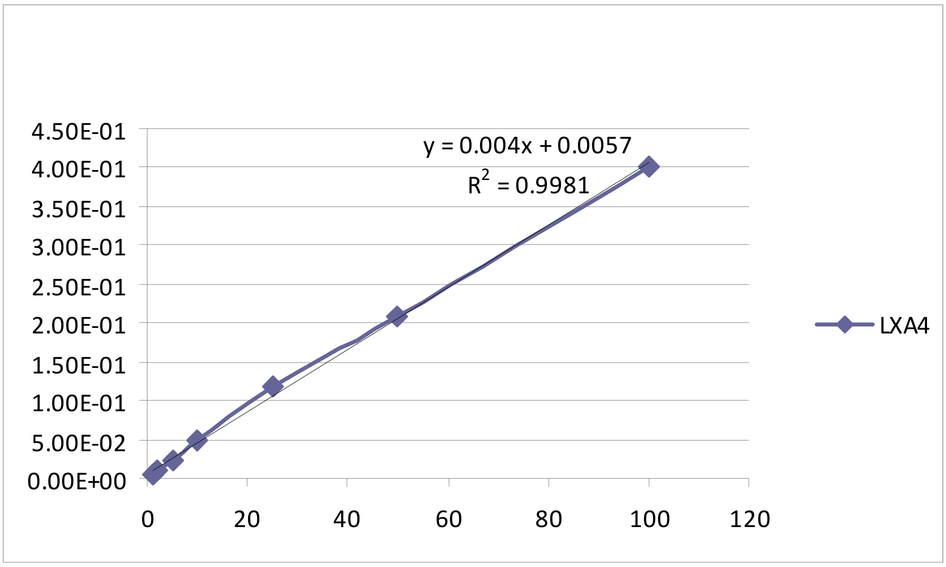

y = 0.004x + 0.0057
R^2 = 0.9981
4.50E-01
4.00E-01
3.50E-01
3.00E-01
2.50E-01
2.00E-01
1.50E-01
1.00E-01
5.00E-02
0.00E+00
0
20
40
60
80
100
120
LXA4

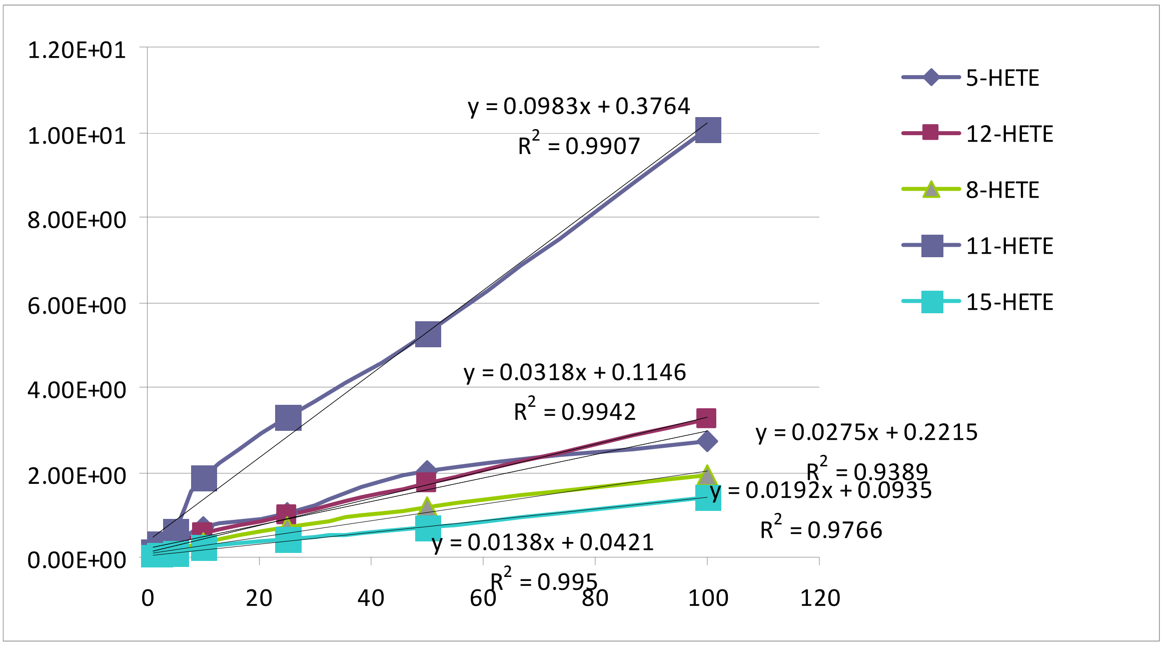
1.20E+01
1.00E+01
8.00E+00
6.00E+00
4.00E+00
2.00E+00
0.00E+00
0
20
40
60
80
100
120
y = 0.0983x + 0.3764
R2 = 0.9907
y = 0.0318x + 0.1146
R2 = 0.9942
y = 0.0275x + 0.2215
R2 = 0.9389
y = 0.0192x + 0.0935
R2 = 0.9766
y = 0.0138x + 0.0421
R2 = 0.995
5-HETE
12-HETE
8-HETE
11-HETE
15-HETE

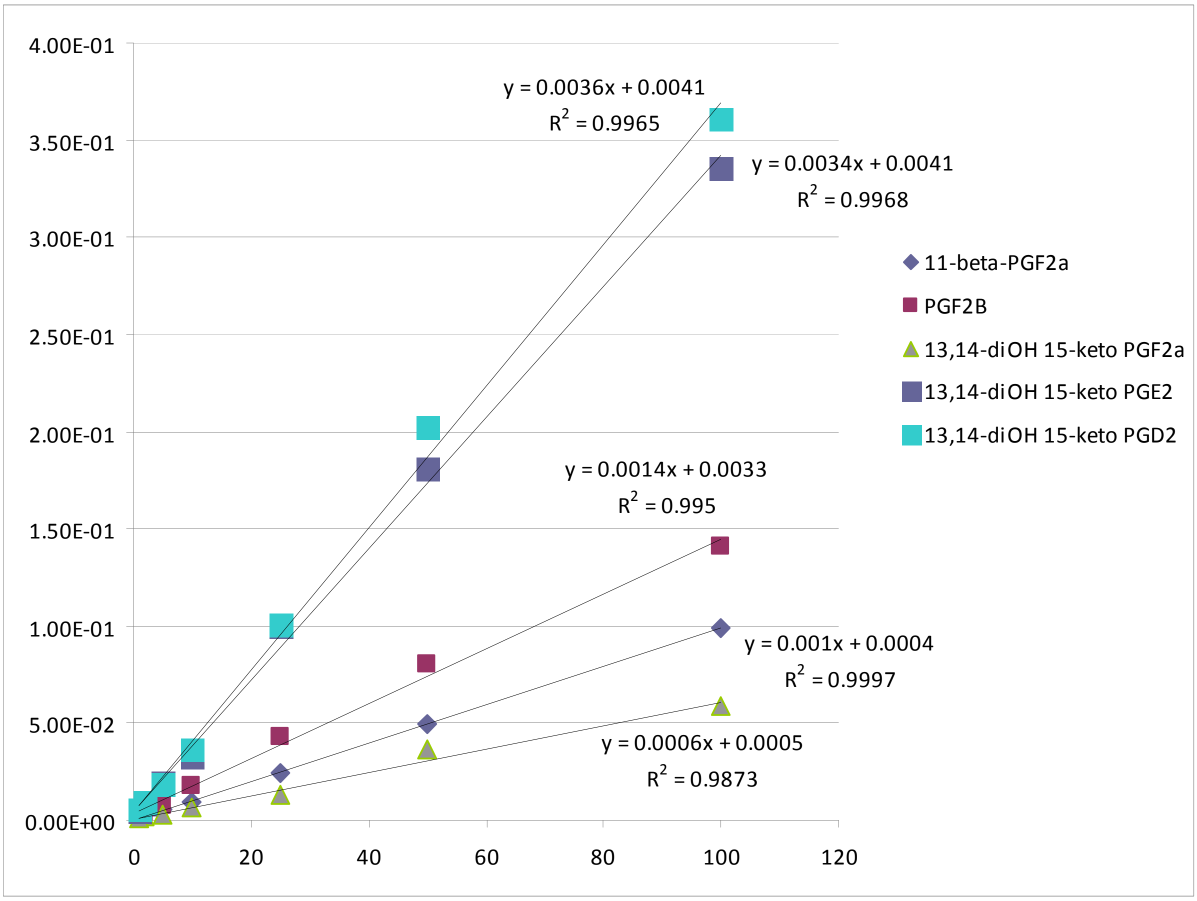
4.00E-01
3.50E-01
3.00E-01
2.50E-01
2.00E-01
1.50E-01
1.00E-01
5.00E-02
0.00E+00
0
20
40
60
80
100
120
y = 0.0036x + 0.0041
R2 = 0.9965
y = 0.0034x + 0.0041
R2 = 0.9968
y = 0.0014x + 0.0033
R2 = 0.995
y = 0.001x + 0.0004
R2 = 0.9997
y = 0.0006x + 0.0005
R2 = 0.9873
11-beta-PGF2a
PGF2B
13,14-diOH 15-keto PGF2a
13,14-diOH 15-keto PGE2
13,14-diOH 15-keto PGD2

**Mediator**	**R^2**	**rho**
15d12,14PGJ2	0.9971	0.998549
PGJ2	0.9918	0.995892
PGD2	0.9991	0.99955
PGE2	0.999	0.9995
PGF2a	0.9981	0.99905
PGE1	0.9981	0.99905
6ketoPGF1a	0.9941	0.997046
TXB2	0.9965	0.998248
13,14diOH15ketoPGE2	0.9968	0.998399
LTE4	0.9944	0.997196
LTD4	0.9964	0.998198
LTB4	0.985	0.992472
LXA4	0.9981	0.99905
5hete	0.9389	0.968969
8hete	0.9766	0.988231
11hete	0.9907	0.995339
12hete	0.9942	0.997096
15hete	0.995	0.997497
13,14diOH15ketoPGD2	0.9965	0.998248
13,14diOH15ketoPGF2a	0.9873	0.99363
PGF2b	0.995	0.997497
11b-PGF2a	0.9997	0.99985
